# Benign adenomyoepithelioma of the breast: a case report

**DOI:** 10.11604/pamj.2022.41.7.28654

**Published:** 2022-01-04

**Authors:** El Habib Belhaddad, Sara Ait Souabni, Khadija Nejmaddine, Ihsane Oubahha, Abderrahim Aboulfalah, Abderraouf Soummani

**Affiliations:** 1 Faculty of Medicine and Pharmacy of Marrakesh, Cadi Ayyad University, Marrakesh, Morocco,; 2 Department of Obstetrics and Gynecology, University Hospital Mohammed VI, Marrakesh, Morocco

**Keywords:** Adenomyoepithelioma, breast lump, breast tumor, adenomyoepitheliomas (AMEs), case report

## Abstract

The diagnosis of adenomyoepitheliomas is difficult and relies on the presence of a double component of epithelial and myoepithelial cells belonging to the breast lobules and ducts. The clinical and imaging characteristics are not specific; thus, the diagnosis is histological. In this article, we present a case of a young female who presented with a 2 cm lump in the breast without other clinical symptoms, which revealed a benign adenomyoepithelioma (AME). We performed a large excisional lumpectomy, and the patient recovered well with no complication or recurrence within two years follow-up. When it comes to adenomyoepitheliomas, the published literature is mainly composed of case reports, so much so that there are no evidence-based guidelines. Our case shows that an excisional lumpectomy is often enough when facing a small size tumor with no signs of malignancy, which contributes to the limited data on the subject.

## Introduction

Among breast tumors, AMEs represent one of the rarest entities. Most of them are benign in nature and have a good prognosis, but sometimes they may show malignant transformation and give recurrences or even metastasis. AMEs are usually difficult to diagnose and need an experimented pathologist and the using of immunohistochemistry. Sometimes they can be misdiagnosed when the core needle biopsy doesn´t encompass both the epithelial and myoepithelial components [1]. The management usually consists of a lumpectomy or wide excision if the size is under 3 cm, whereas a mastectomy with axillary lymph node resection is usually performed when the size is bigger [1]. We report an uncommon case of a 34-year-old woman with benign adenomyoepithelioma of the breast and describe our management and the outcome of the patient within two years follow-up.

## Patient and observation

**Patient information:** our patient was a 34-year-old woman with no relevant past medical history, who presented with a nodule of the breast that was accidentally discovered on self-examination. The nodule was located in the inner lower quadrant.

**Clinical findings:** on physical examination, there was a well-limited, painless and mobile breast nodule, located in the inner lower quadrant of the left breast. There were with no inflammatory signs and no palpable lymphadenopathies. The rest of the examination was unremarkable.

**Diagnostic assessment:** on ultrasonography, it was a solitary lump of hypoechogenic texture, located in the inner lower quadrant, with lobulated margins and no calcifications, that could be classified American College of Rheumatology (ACR) 3 or 4. The measurements were approximately 2 cm in length, 2 cm in width and 1.5 cm in thickness. We also performed a core needle biopsy.

**Figure 1:**
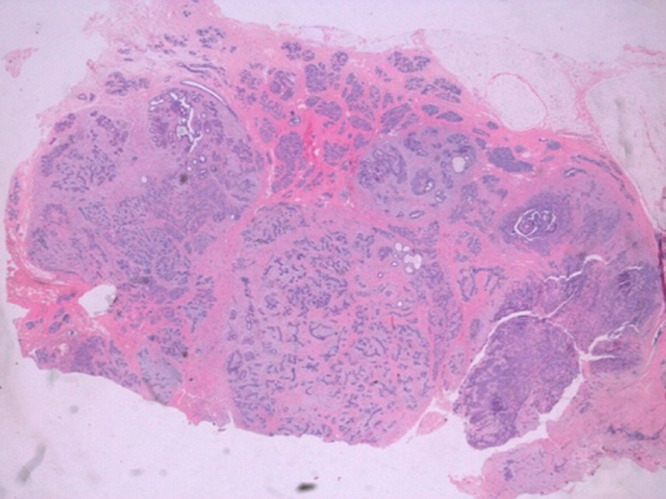
histopathological aspect of adenomyoepithelioma of the breast on low magnification

**Figure 2:**
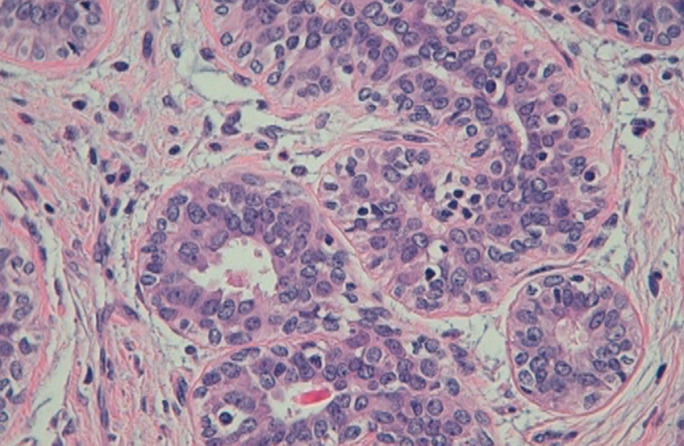
histopathological aspect of adenomyoepithelioma of the breast on high magnification: the inner layer is composed of epithelial cells with eosinophilic cytoplasm, and is bordered with myoepithelial cells

**Diagnosis:** the biopsy showed a double composition of the tumor. We could see both epithelial and myoepithelial cells which was consistent with adenomyoepithelioma (Figure 1, Figure 2). There were no cytological abnormalities nor infiltration and mitotic index was low (Figure 2). Immunochemistry was positive for smooth muscle actin +, p63+, cytokeratin 14+, cytokeratin 5+ (myoepithelial contingent), and AE 1/3 (epithelial contingent). Hormonal receptors (RE, RP) and HER2 were negative.

**Therapeutic interventions:** since the tumor was small in size, and there were no signs of malignancy both clinically and histologically, we decided to perform a large excisional lumpectomy. The pathological report confirmed the adenomyoepithelioma, and the surgical margins were clear.

**Follow-up and outcome of interventions:** we followed up the patient for two years. The first post-operative visit was after 1 month, then every 6 months. There were no post-operative complications, no recurrence and no distant metastasis.

**Informed consent:** the patient provided her full consent after oral explanation of our intention of publishing her case.

## Discussion

Adenomyoepitheliomas are rare breast tumors that are characterized by their double composition of both epithelial and myoepithelial cells. Although they are usually benign, their behavior can be unexpected and they can transform into malignancies, whether originating from the epithelial component, the myoepithelial component or from both of them [2]. It was Hamper in 1970 that first described AMEs [3], then a further subcategorization was done by Tavassoli in 1991, where he proposed a classification system based on myoepithelial lesions of the breast into 4 categories: spindle-cell type, tubular type, lobulated type, and carcinoma arising in adenomyoepithelioma [4]. All of them can coexist in the same tumor.

These tumors can appear at different ages ranging from 16 to 86 years [5], with a median age of 56.75 years [6]. Our patient was only 34 years old. Often, they present in the form of a single breast nodule, with a median tumor size of 2 cm [7]. If there is a rapid progression in size it is strongly suggestive of malignant transformation. Only one case of bilateral adenomyoepithelioma has been described by Bajpai *et al*. in 2013 in a 16-year-old female [8], and two cases of AMEs in male patients have been reported. The first one in 1991 in a 47-year-old patient with history of lymphoma [9], and the second one in 1997 [10]. Both of them were benign.

The radiological images are not specific. Typically, on mammogram, they present as round, oval or lobulate high density masses with sharp bordures [11]. Sometimes there can be indistinct margins [12]. The size varies between 0.3 to 7 cm with an average diameter of 2.5 cm. A bigger size and irregular borders can be potential signs of malignancy. Microcalcifications are rare but have been reported, and have poor outcome. On magnetic resonance imaging (MRI), AMEs usually present as isointense masses on T1WA and appear hyperintense on T2W1, with a homogeneous progressive enhancement [11], or heterogeneous enhancement with washout or plateau enhancement kinetics [12].

On a pathological perspective, the diagnosis of AMEs is difficult on core biopsy. Once it is suspected, the immunochemistry confirms it by bringing out the characteristics of each component apart: 1) The myoepithelial part is shown by the positivity of cytokeratin 5/6 antibodies, calponin, p63, smooth muscle actin, smooth muscle myosin, caldesmone, cd10 and S100 protein; 2) the epithelial part shows a positive staining for low molecular weight keratin, cytokeratin antibodies and AE1/AE3 [13].

P53 and KI-67 are known to be prognostic factors in AMEs. When positive, they yield a poorer outcome [14]. Three pathological malignancy criteria have been identified by Loose *et al*. in 1992: high mitotic activity (>3 mitosis per high-power-field x400), cytonuclear abnormalities and infiltration [15]. The main differential of the tubular subtype is microglandular adenosis; the absence of myoepithelial base can help distinguishing them. Other differentials are tubular adenoma, papilloma and leiomyoma [16].

Whether benign or malignant, AMEs can reoccur. Positive margins or narrow margins are predictive. If narrow margins or incomplete margins are detected, a second excision is needed [17]. Recurrences have been described between 4 months or as late as 23 years [5]. The spreading to other sites is possible and have been seen in the lungs, liver, brain, thyroid, soft tissue and bone; suggesting that there is more likely a hematogenous spreading rather than a lymphatic one [17]. That is why in the management of AMEs, axillary lymph node dissection is usually not indicated. However, rare cases of lymph node metastasis have been described (sometimes without palpable lymphadenopathy), suggesting that sentinel lymph node sampling is needed in case of malignant adenomyoepithelioma [18].

The use of chemotherapy, radiotherapy and hormonal therapy have not shown that much success [13]. In a recent case series using a large US database where 110 cases have been analyzed, chemotherapy was used in 26% of cases, hormonal therapy in 8% and radiotherapy in 36%, without improvement of overall survival (OS) in that population [7].

## Conclusion

The management of AMEs is not subject to clear guidelines due to its rarity. But reviews have shown that large excisional lumpectomy is usually enough for the benign forms, which account for the majority of cases, including ours. On the other hand, it seems more appropriate to perform a mastectomy in malignant forms. Sentinel lymph node analysis is debatable, because the spreading to lymphatic nodes is extremely rare but has been described, and systemic therapies don´t seem to be effective.
